# Infectious Disease Mortality Rates, Thailand, 1958–2009

**DOI:** 10.3201/eid1811.120637

**Published:** 2012-11

**Authors:** Suchunya Aungkulanon, Margaret McCarron, Jongkol Lertiendumrong, Sonja J. Olsen, Kanitta Bundhamcharoen

**Affiliations:** International Health Policy Program (IHPP), Nonthaburi, Thailand (S. Aungkulanon, J. Lertiendumrong, K. Bundhamcharoen);; Centers for Disease Control and Prevention, Atlanta, Georgia, USA (M. McCarron, S.J. Olsen);; and Thailand Ministry of Public Health–United States Centers for Disease Control and Prevention Collaboration, Nonthaburi (S.J. Olsen)

**Keywords:** infectious disease, mortality rates, epidemiologic transition, bacteria, viruses, parasites, Thailand

## Abstract

To better define infectious diseases of concern in Thailand, trends in the mortality rate during 1958–2009 were analyzed by using data from public health statistics reports. From 1958 to the mid-1990s, the rate of infectious disease–associated deaths declined 5-fold (from 163.4 deaths/100,000 population in 1958 to 29.5/100,000 in 1997). This average annual reduction of 3.2 deaths/100,000 population was largely attributed to declines in deaths related to malaria, tuberculosis, pneumonia, and gastrointestinal infections. However, during 1998–2003, the mortality rate increased (peak of 70.0 deaths/100,000 population in 2003), coinciding with increases in mortality rate from AIDS, tuberculosis, and pneumonia. During 2004–2009, the rate declined to 41.0 deaths/100,000 population, coinciding with a decrease in AIDS-related deaths. The emergence of AIDS and the increase in tuberculosis- and pneumonia-related deaths in the late twentieth century emphasize the need to direct resources and efforts to the control of emerging and re-emerging infectious diseases.

## Abstract 2

Reliable, relevant, and timely data guide public health policies that protect and promote health.

Infectious diseases were responsible for a considerable number of deaths in Thailand during the mid–twentieth century (*1*). During 1948–1955, as Thailand experienced substantial economic and social development and transitioned from an agricultural to an urban and industrial society (*2*), the mortality rate began to decline (*3*). In 1968, infectious diseases such as tuberculosis (TB) and pneumonia were the main cause of death in Thailand; fewer deaths were caused by noninfectious diseases (e.g., diseases of the heart, malignancies). However, in the early 1980s, an epidemiologic transition was taking place, and non-communicable diseases became of greater public health concern (*4*). In contrast with the low mortality rates in many industrialized countries, where communicable diseases are well controlled (*5*,*6*), mortality rates in Thailand remained relatively high. However, the emergence of HIV/AIDS contributed to an increase in deaths in the 1990s and interrupted the epidemiologic transition (*7*,*8*).

Communicable diseases are still responsible for a considerable number of illnesses (10% of total diseases in 2009) and deaths in Thailand (*9*). Because of the increasing threat from emerging and reemerging infectious diseases, it is vital to understand the patterns of infectious disease–related deaths in a country that has undergone economic development and concurrent improvements in health, sanitation, and access to healthcare. We used publicly available vital statistics for deaths in Thailand to analyze trends in infectious disease mortality rates during 1958–2009. This assessment helps us better understand past trends and inform policy on current infectious diseases of public health concern.

## Methods

### Source of Data

Death-related data were obtained from a published series called the Report of Public Health Statistics (Bureau of Policy and Strategy, Ministry of Public Health [MOPH], Nonthaburi, Thailand). The reports summarize data, by year, from death certificates provided by the MOPH in collaboration with the Ministry of Interior (MOI) as part of the Vital Registration System. The MOI is responsible for registering deaths at the local administrative level; the MOPH is responsible for processing vital statistics data for the whole country and for disseminating the information on an annual basis through publication of the Report of Public Health Statistics. Death certificates record only 1 cause of death, which is the underlying cause of death.

Using data from 1958–2009, we created an electronic database from the series of Report of Public Health Statistics. The database provided aggregated information on the total number and rate of deaths by age group, sex, year of death, and cause of death. Cause of death was coded according to the International Classification of Disease (ICD). During 1958–2009, the following ICD revisions were used: 1958–1967, ICD version 7 (ICD-7); 1968−1976, ICD-8; 1977–1993, ICD-8; and 1994–2009, ICD-10. For each ICD revision, death data were grouped differently, resulting in different group numbers for the ICD versions: ICD-7, 50 groups; ICD-8, 150; ICD-9, 56; and ICD-10, 103. During 1958–1983, mortality rates were calculated by using population denominator data from the Population and Housing Censuses conducted in 1960, 1970, and 1980. The estimated population between census years was adopted from the Report of the Working Group on Population Projection. After 1984, the annual mid-year estimated population was obtained from the Bureau of Registration Administration, MOI.

### Determination of Infectious versus Noninfectious Disease

To assess trends in deaths caused by infectious diseases, we applied a classification scheme developed at the Centers for Disease Control and Prevention (CDC, Atlanta, GA, USA) (*6*). This coding system used ICD-9 codes to classify infection-associated diseases as 1) an infectious disease (e.g., pneumonia), 2) possibly an infectious disease (e.g., pityriasis rosea), and 3) result of an infectious disease (e.g., rheumatic fever). The system was developed to address the exclusion of some infectious diseases from the infectious disease category of the ICD system, e.g., influenza, which was placed in the respiratory disease category. For the purposes of this study only, those diseases classified as an infectious disease or as the result of an infectious disease (*1*,*3*) were used to classify deaths from infectious disease.

Several adjustments were made to the original coding system. First, we had to account for deaths that were reported by using grouped ICD codes without distinction for infectious versus noninfectious disease (e.g., bronchitis, emphysema, and asthma are grouped in ICD-7). Deaths were reported by using only the shorter, less detailed 3-digit codes rather than the 4-digit subcodes used in the CDC classification system; thus, we re-coded as infectious any 3-digit codes for which >80% of the subcodes were for infectious diseases, and we re-coded as noninfectious any 3-digit codes for which <80% of the subcodes were for infectious diseases.

Second, we classified deaths during 1994–2009 (reported with ICD-10 codes) by using the CDC system and assigning the infectious determination of their correlated ICD-9 code. These classifications facilitated an analysis of overall trends in deaths caused by infectious versus noninfectious diseases.

Third, we specifically excluded deaths from septicemia, which were first reported in the series of Report of Public Health Statistics in 1994, when use of ICD-10 coding began. Septicemia accounted for ≈15% of in-hospital deaths in vital registration data, but after verbal autopsy was used to validate cause of death data, septicemia was determined to cause <1% of deaths; thus, it was decided that deaths due of septicemia should be largely reassigned to cerebrovascular disease (*10*). To avoid misclassification, deaths from septicemia from 1994 forward were removed from the infectious disease category.

To address the problem posed by the frequent revisions of the ICD coding system, we used ICD-9 as the standard and recoded deaths that were reported by using other ICD versions. The codes used for all deaths reported by using ICD-7 and -8 were converted to ICD-9 codes by using proper disease names as defined, respectively, in each version of the ICD system. The conversion from ICD-10 to ICD-9 codes was done using a published tool developed by the American Academy of Professional Coders (*9*). A complication in the conversion backward from ICD-10 to ICD-9 was the presence of several many-to-one and one-to-many coding relationships, caused by a significant change in the detail of diagnostic codes; this change resulted in a near doubling of the number of diagnostic codes (*11*). Once uniform coding was established for all deaths, the infectious disease coding system described above was applied to these deaths.

### Determination of Specific Infectious Categories

The cause-of-death data from the series of Public Health Statistics were reported according to the ICD tabulation list, which differed among the ICD revisions. These lists provide a short set of aggregate codes intended to facilitate cause-of-death reporting in countries with more limited capacity. We consistently identified 8 categories of infectious diseases for analysis as separate categories: TB, gastrointestinal infection, HIV/AIDS, pneumonia, sexually transmitted diseases, diphtheria, polio, and malaria (Table).

**Table:** ICD codes for 8 groups of infectious disease groups consistently found in a study of trends in infectious disease mortality rates, Thailand, 1958–2009*

Disease	ICD-7, 1958–1967	ICD-8, 1968–1976	ICD-9, 1977–1993	ICD-10, 1994–2009
Malaria	110–117	84	080–088	B50–B54
Tuberculosis	001–008, 010–019	010–019	010–018	A15–A19
HIV/AIDS	NA	NA	NA	B20–B24
Pneumonia	490–493	480–486	480–486	J12–J18
Gastrointestinal infection	040, 043, 045–048	000–004, 006, 008, 009	001–009	A00–A09
Sexually transmitted infection	020–029	090–098	090–099	A50–A64
Diphtheria	055	032	032	A36
Polio	080	040, 043, 044	040, 043, 044	A80

*ICD, International Classification of Disease; NA, not applicable.

### Analysis of Trends

Multiple period regression was used to estimate the difference of the magnitude of trend in mortality rate. Average annual rate of change and 95% CIs are presented. All-cause deaths were age-adjusted by 5 age groups (0–4, 5–24, 25–44, 45–64, and >65 years); because of the structure (aggregate data) of the database, it was not possible to age-adjust other rates.

## Results

### Overall Mortality Rate Trends

**Figure 1:**
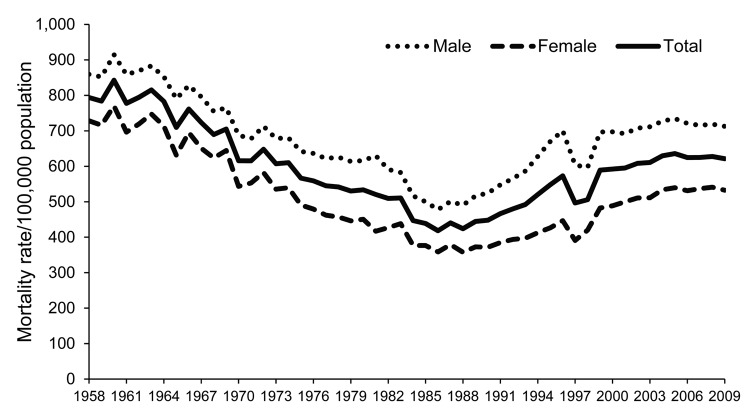
All-cause mortality rates, Thailand, 1958–2009.

The all-cause mortality rate during 1958–2009 was characterized by a decrease during 1958–1986 and an increase during 1987–2009; however, in the early 2000s, the rate leveled (Figure 1). The average annual decrease for 1958–1986 was 2.8 deaths/100,000 population (95% CI 14.1%–11.5%) and average increase for 1987–2009 was 10.8 deaths/100,000 population (95% CI 9.3%–12.4%). The pattern was similar for both sexes, but the annual rate for males consistently exceeded that for females (Figure 1).

**Figure 2:**
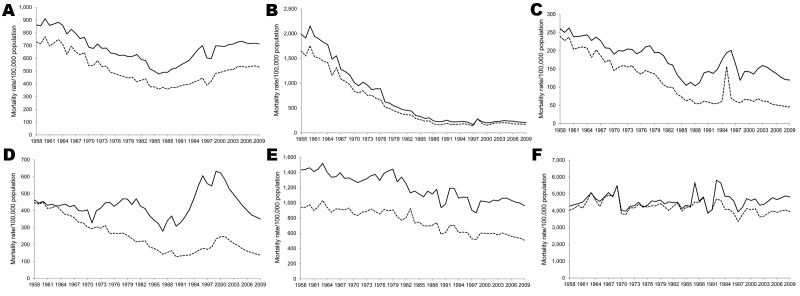
All-cause mortality rates, by age group and sex (solid lines, male; dashed lines, female), Thailand, 1958–2009. A) All ages; B) 0–4 years of age; C) 5–24 years of age; D) 25–44 years of age; E) 45–64 years of age; F) >65 years of age.

The all-cause mortality rate varied by age group, but it was among persons >65 years of age (Figure 2). The highest average annual decline in the mortality rate was among children <5 years of age; the decline (34.3 deaths/100,000 population; 95% CI 26.2% –42.4%) represented a 10-fold reduction from 1,820.1 deaths/100,000 population in 1958 to 188.1/100,000 in 2009. From the 1990s through 2009, there was almost no difference between the mortality rate for boys and that for girls (Figure 2). During this same period, all-cause mortality rates among persons 5–24 years of age declined 3-fold from 250.0 deaths/100,000 population in 1958 to 83.5/100,000 in 2009. For persons 5–64 years of age, the mortality rate was higher for males than females. The difference in the mortality rate between sexes was most pronounced for persons 25–44 years of age, especially during 1996, when there was a 3-fold difference for males (605.9 deaths/100,000 population) versus females (178.4 deaths/100,000 population). All-cause mortality rates in persons 5–24 and 25–44 years of age increased during 1990–2000.

### Infectious Disease Mortality Rate Trends

**Figure 3:**
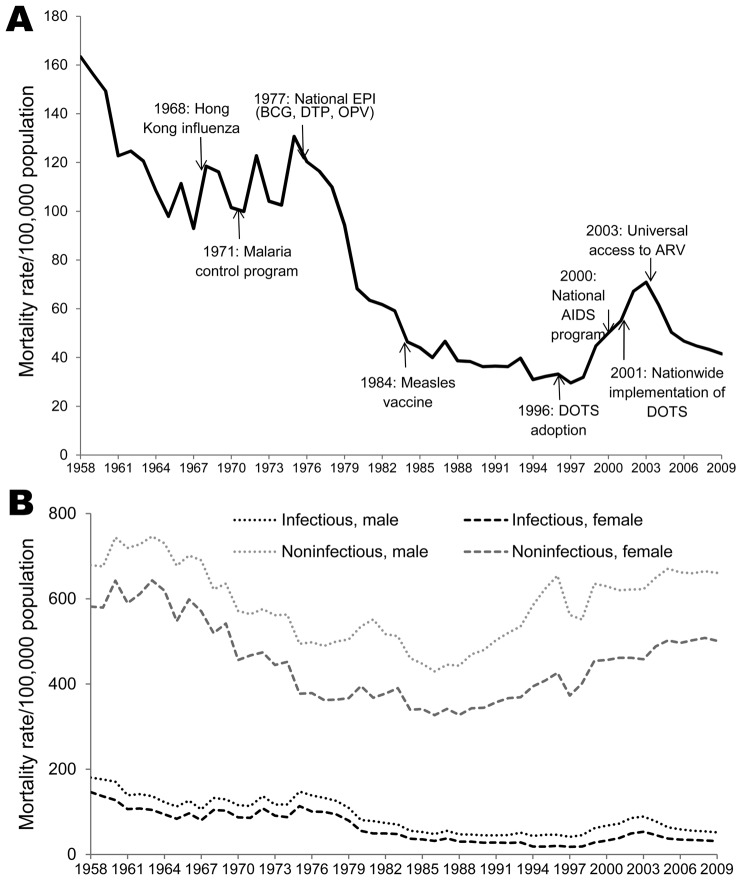
Mortality rates for infectious and noninfectious diseases, Thailand, 1958–2009. A) Infectious disease–related mortality rates, major events, and key public health interventions. B) Comparison of infectious disease–related mortality rates with noninfectious disease–related mortality rates. EPI, Expanded Program on Immunization; BCG, Bacillus Calmette–Guérin vaccine; DTP, diphtheria, tetanus, and pertussis vaccine; OPV, oral polio vaccine; ARV, antiretroviral treatment; DOTS, directly observed treatment, short course.

From 1958 through the late 1990s, the infectious disease mortality rate in Thailand declined 5-fold, from 163.4 deaths/100,000 population in 1958 to 29.5/100,000 in 1997 (average annual reduction 3.2 deaths/100,000 population; 95% CI 2.8%–3.7%) (Figure 3). This decline paralleled the decline in overall deaths from 1958 to the late 1990s. In 1998, infectious disease–related mortality rates started to increase and the trend continued through 2003 (average annual increase 7.6 deaths/100,000 population; 95% CI 5.9%–9.4%). In 2004, infectious disease–related deaths began to decline again; during 2004–2009, the average annual reduction was 3.5 deaths/100,000 population.

**Figure 4:**
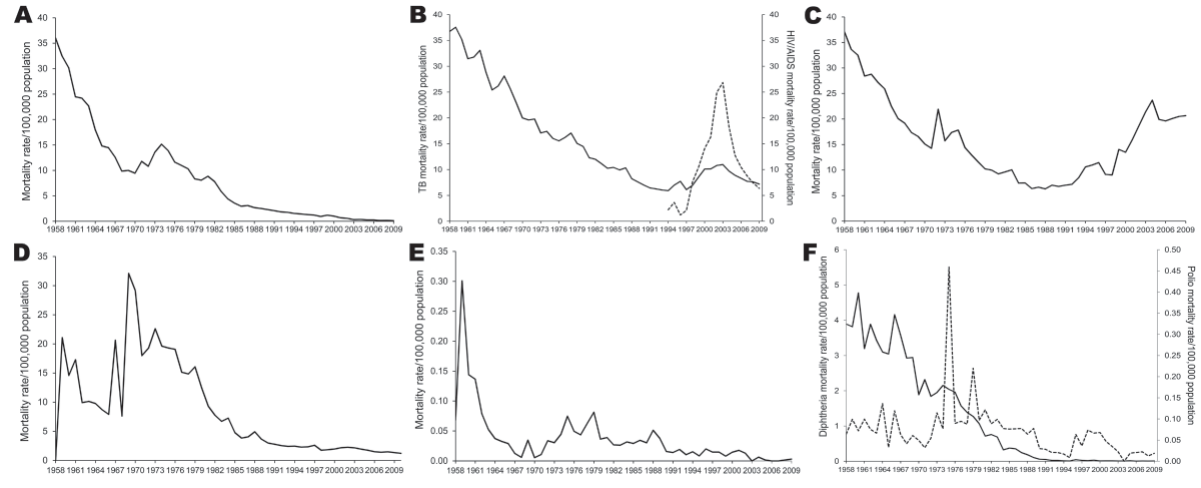
Infectious disease–related mortality rates for select diseases, Thailand, 1958–2009. A) Malaria; B) tuberculosis and HIV/AIDS; C) pneumonia; D) gastrointestinal infection; E) sexually transmitted infections; F) diphtheria and polio.

Mortality rates for several specific infectious diseases declined during 1958–2009 (Figure 4). For example, malaria deaths declined from 36.0/100,000 population in 1958 to 0.1/100,000 population in 2009. Diphtheria-related deaths decreased throughout the study period. In contrast, deaths from polio showed much year-to year variability, with a surge in 1975; however, polio-related deaths remained low (<20 deaths/year) during 2002–2009. Mortality rates for gastrointestinal infection fluctuated through the 1960s but then declined rapidly.

Three phases characterized TB-related mortality rates: 1958–1994, 1995–2003, and 2004–2009 (Figure 4). During the first phase, rates sharply declined from 36.7 deaths/100,000 population in 1958 to 5.9/100,000 in 1994. During the second phase, the trend deaths reversed, increasing from 7.0 deaths/100,000 population in 1995 to 11.0/100,000 in 2003. During the third phase, TB-related mortality rates again declined, decreasing from 9.7 deaths/100,000 population in 2004 to 7.2/100,000 in 2009. Deaths from HIV/AIDS, first reported in the series of Report of Public Health Statistics in 1994, peaked at 26.8/100,000 population in 2003 but declined to 6.4/100,000 in 2009 (Figure 4).

Pneumonia-related mortality rates, similar to those for TB, decreased from 37.0 deaths/100,000 population in 1958 to 7.0/100,000 in 1991 and then increased sharply (Figure 4). The increasing trend in pneumonia-related deaths, beginning in 1993, was more pronounced among persons 25–44 and >60 years of age (data not shown).

During 1959–1968, mortality rates for sexually transmitted diseases dropped sharply. However, starting in the early 1970s, the rates increased for a decade before declining again (Figure 4).

## Discussion

Our findings demonstrate a substantial decrease in deaths overall in Thailand from 1958 through 1986, followed by an increase beginning in 1987 and then a leveling-off beginning in the early 2000s. All-cause mortality rate trends were similar for males and females, but the rate was consistently higher for males. The gender gap in all-cause mortality rates among persons 25–44 years became more pronounced in recent years, mostly because of fatal traffic accidents and HIV/AIDS-related deaths among men, a group that was more affected by HIV/AIDS in the first phase of the epidemic (*12*,*13*). Age-specific mortality rates for children 0–4 years of age showed the greatest decline; the development of and equitable access to maternal and child health care services and vaccination may have contributed to this decline (*14*,*15*). The all-cause mortality rate for children 0–4 years of age declined over the study period; however, after the introduction of the Expanded Program on Immunization (EPI), the rate declined 28% (from 715 to 514 deaths /100,000 population). The extensive geographic coverage of primary health care services contributed significantly to maternal and child health outcomes (*16*).

From 1958 through the mid-1990s, the infectious disease mortality rate in Thailand declined substantially, largely because of declines in malaria, TB, pneumonia, and gastrointestinal infection. Several factors contributed to this trend, including general improvements in sanitation, improved access to medical care (a result of health infrastructure expansions at the district level), and financial risk protection (*16*), and the introduction of routine childhood vaccination through EPI, which was officially launched in 1977 (*14*). Since 1987, coverage with TB, diphtheria-tetanus-pertussis, and tetanus toxoid vaccines has been 96%, 75%, and 60%, respectively (*17*). Deaths related to vaccine-preventable infectious disease declined sharply in association with >90% EPI coverage in the 1990s (*18*). In the United States, studies have shown a decline in infectious disease–related deaths during the twentieth century (*5*,*6*).

In the late 1990s, the decreasing trend for the infectious disease–related deaths reversed. Disease categories that contributed most to this reversal were HIV/AIDS, TB, and pneumonia; all of which had sharply elevated mortality rates during 1997–2003 and decreasing rates in 2004. HIV/AIDS emerged in Thailand in the mid-1980s and spread rapidly with devastating effects (*19*). In 1999 in Thailand, HIV/AIDS had become the leading cause of death in men 25–44 years of age, resulting in a widening gap in the mortality rate between men and women (*8*,*13*). Co-infection with TB and HIV is common; thus; the rising number of TB-related deaths during 1995–2003 coincided with the explosive epidemic of HIV infection and gradually led to the emergence of multidrug-resistant TB (*20*,*21*). Moreover, the reported incidences of pneumonia and pneumonia-related deaths had been increasing in Thailand since the mid-1980s (*22*). It is likely that HIV/AIDS contributed to the pneumonia-related mortality rate. However, this contribution was not readily apparent until later because HIV/AIDS was not a reportable cause of death until 1994 and because stigma was likely a barrier to reporting in the early years of the HIV/AIDs epidemic (*23*). Furthermore, if the code for opportunistic infectious diseases was used for deaths caused by HIV/AIDS, the number of deaths from HIV/AIDs may have been underestimated (*10*,*24*). In persons with HIV/AIDS in Southeast Asia, the common opportunistic infections were TB, cryptococcosis, and *Pneumocystis carinii* and *Pennicillium marneffei* fungal infections, all of which can cause pneumonia (*25*). The observed increase in pneumonia-related deaths in very elderly persons may also reflect the misclassification of the cause of death because pneumonia is often the immediate, rather than underlying, cause of death. A study to verify cause of death data found that ≈31% of deaths coded as being caused by pneumonia should have been classified as being caused by cerebrovascular disease, chronic obstructive pulmonary disease, diabetes, or genitourinary disease (*10*).

We found that deaths from HIV/AIDS and TB declined during 2004–2009; this decline may account for the concurrent leveling-off of infectious disease–related mortality. Thailand implemented a national AIDS program in 1991 and a national antiretroviral (ARV) treatment program in 2000. The ARV treatment program was designed to increase access to health care and treatment, but it was not until 2004 that the universal ARV treatment program was fully implemented, providing ARV treatment for all eligible patients under the National Access to ARVs for People Living with HIV/AIDS program. As the program scaled up, universal ARV contributed significantly to the reduction in AIDS-related deaths (*26*–*28*). Two parallel prevention programs, vertical transmission prevention and condom promotion, contributed to decreasing the incidence of HIV infection and changed the epidemic in Thailand from one that was generalized to one that was concentrated in certain subpopulations, particularly injection-drug users, homosexual men, and youth. TB-related deaths were also reduced through early initiation of ARV treatment program for persons with HIV, implementation of DOTS (directly observed treatment, short course), and appropriate antimicrobial drug regimens for TB treatment (*29*).

DOTS was implemented nationwide in Thailand in 2001, and since then, the country has achieved the international goal for detecting >70% of the estimated cases of infectious TB (i.e., cases in persons with a TB-positive sputum smear); however, Thailand has not met the international goal for successfully treating >85% of the detected cases (*30*). The main issues relating to TB control were the fragmented delivery of services; limited capacity of stakeholders and limited coordination between stakeholders; weak referral linkages between hospitals and health centers; high TB/HIV co-infection rates and limited access to ARV treatment, especially among poor persons and those with less education; unsupervised treatment with high default rates; and widespread unregulated use of second-line antimicrobial drugs, which could lead to an outbreak of multidrug-resistant TB and extensively drug-resistant TB (*31*). Certain infectious diseases (e.g., TB) are re-emerging, and emerging HIV/AIDS epidemics in certain subpopulations have considerable implications for the Thai population. Continued monitoring and evaluation of the effect of interventions on disease incidence and mortality rates are critical if the global goal of curing 85% of TB cases is to be achieved.

We reviewed mortality rates during 1958–2009 in Thailand by using a standardized infectious disease classification scheme. Three possible artifacts in year-to-year fluctuations in the mortality rate over the study period should be considered. The first artifact concerns changes that were made to Thailand’s data recording system during the study period. Four versions of the ICD systems were used, and ICD code changes can lead to substantial changes in long-term trends in cause-specific mortality rates (*32*). Our results show an increasing trend in deaths from pneumonia and TB since 1994, when the switch was made from ICD-9 to ICD-10 coding; we have not accounted for the differences in diagnostic trends concurrent with the changes in coding. When a US National Center for Health Statistics comparability ratio was applied to the US mortality rates for influenza and pneumonia, it appeared that the declines mainly resulted from the introduction of the ICD-10 coding system (*33*). We did not apply comparability ratios in our analysis because such ratios were not developed with the data provided by the Thai MOPH.

The second possible artifact is that deaths sharply declined from 1996 to 1997, and this decline was followed by a sharp increase from 1998 to 1999. We cannot rule out that such sudden changes may have resulted from changes in the process for reporting deaths, which was implemented in 1996. In the new death certification system, each death was entered into the computer database in the Civil Registration Database at the Bureau of Registration Administration of the MOI and then transferred to the vital registration database at the MOPH.

The third possible artifact is the cause of death coding errors. The coding of polio deaths since 1997, is an example of such errors. The Polio Eradication Campaign in Thailand was started in 1990, and the last polio case was reported in April 1997 (*34*). Cases of and deaths from acute flaccid paralysis are aggressively investigated, making it unlikely that a single death could be missed. However, despite the lack of any reported cases of polio since 1997, a total of 274 polio-related deaths were coded during 1997–2009. In general, the quality of death statistics in Thailand is considered poor because many death registrations are incomplete and a large proportion have poorly defined causes of death (*11*,*35*). Furthermore, during the 1960s and 1970s, only ≈60% of deaths were registered. That percentage increased to 76% in the mid-1980s and to 95% in the mid-1990s. The highest proportion of unregistered deaths was for infants; this was a result of death occurring, in many cases, before the birth was registered (*4*,*36*). The proportion of in-hospital deaths increased from 20% during the 1980s to 43% in 2009 (*37*). The validity of the overall cause-of death statistics during that time is questionable because many out-of-hospital deaths were coded by persons not medically qualified to determine the cause of death. All of these factors may have influenced the study findings.

This study has some possible limitations. The analysis was based on death attributed to the underlying cause of death as it was reported on death certificates and published in the Report of Public Health Statistics. Because death records list only one cause of death, we knew only the underlying cause of death and, thus, may have underestimated the effect of infectious diseases as a contributing cause of death. As a result, this study may underestimate the role of infectious diseases on mortality rates. In addition, the aggregate nature of our data prevented additional exploration of the specific cause of death by age group. Our estimate of the extent of infectious disease is conservative, focusing exclusively on deaths. This focus reflects only part of the effects from disease because infectious disease may result in substantial illness or disability, or both, without causing death. Future analyses of other dimensions of the effects of infectious diseases, such as their effect on the economy, the number of hospitalizations, and the number of life-years lost because of disability, will provide information to inform policy-making decisions.

The implementation of a malaria control program and new and effective antimicrobial drugs for treating TB contributed considerably to the reduction in communicable disease–related illness and deaths in the second half of the twentieth century (*1*,*38*–*40*). The emergence of HIV/AIDS and the increase in TB- and pneumonia-related deaths in the late twentieth century dramatically interrupted the epidemiologic transition in Thailand. A universal ARV treatment program has rapidly scaled up and resulted in a decrease in the number of deaths from HIV/AIDS after 2003; however, this decline remains unstable. Recent trends emphasize the dynamic process of infectious diseases and highlight the need for sustained resources and efforts to combat their emergence or re-emergence. These data also highlight the importance of addressing health disparities between men and women. Reliable, relevant, and timely mortality data are crucial to guide effective policy responses to protect and promote population health.
